# Secondary metabolites and biodiversity of actinomycetes

**DOI:** 10.1186/s43141-021-00156-9

**Published:** 2021-05-12

**Authors:** Manal Selim Mohamed Selim, Sayeda Abdelrazek Abdelhamid, Sahar Saleh Mohamed

**Affiliations:** Microbial Biotechnology Department—Genetic Engineering Division, National Research Centre, Giza, Egypt

**Keywords:** Actinomycetes, Biodiversity, Bioactive compound, Secondary metabolites

## Abstract

**Background:**

The ability to produce microbial bioactive compounds makes actinobacteria one of the most explored microbes among prokaryotes. The secondary metabolites of actinobacteria are known for their role in various physiological, cellular, and biological processes.

**Main body:**

Actinomycetes are widely distributed in natural ecosystem habitats such as soil, rhizosphere soil, actinmycorrhizal plants, hypersaline soil, limestone, freshwater, marine, sponges, volcanic cave—hot spot, desert, air, insects gut, earthworm castings, goat feces, and endophytic actinomycetes. The most important features of microbial bioactive compounds are that they have specific microbial producers: their diverse bioactivities and their unique chemical structures. Actinomycetes represent a source of biologically active secondary metabolites like antibiotics, biopesticide agents, plant growth hormones, antitumor compounds, antiviral agents, pharmacological compounds, pigments, enzymes, enzyme inhibitors, anti-inflammatory compounds, single-cell protein feed, and biosurfactant.

**Short conclusions:**

Further highlight that compounds derived from actinobacteria can be applied in a wide range of industrial applications in biomedicines and the ecological habitat is under-explored and yet to be investigated for unknown, rare actinomycetes diversity.

## Background

The inquiry and discovery of novel microorganisms that produce new secondary metabolites can be required to stay critical in the race against new and rising diseases and antibiotic-resistant pathogens [1, 2]. Actinomycetes are broadly distributed in natural and man-made conditions and assume a vital role in organic matter degradation. They are additionally notable as a rich source of bioactive secondary metabolites [1]. Secondary metabolites are known as organic compounds which are not specifically associated with the normal growth of an organism, improvement, or propagation of it. The diversity of Actinomycetes and their capacity to produce novel substances put this class in a noticeable position. They are in charge of the generation of about 50% of the explored bioactive secondary metabolites, remarkably antibiotics, anticancer agents, anti-inflammatory agents, and enzymes [3, 4]. In light of the amazing reputation of actinomycetes, a lot of achievement has been centered on the fruitful isolation of new actinomycetes from various sources for medication screening programs in the past 50 years [5]. In the previous two decades, there has been a decrease in the revelation of new critical compounds from basic soil derived actinomycetes where they have produced huge numbers of previously described secondary metabolites [6, 7]. Consequently, this prompts increment in the finding of new actinomycete taxa from abnormal environments which thus prompts make new age of drug specialists [8]. Actinomycetes produce a wide array of biologically active compounds such as antibiotics, enzymes, and enzyme inhibitors [9, 10]. As of late, the rate of discovery of new compounds from actinomycetes of terrestrial sources has been diminished, while the rate of re-isolation of known compounds has likewise been expanded. Consequently, it is significant that new actinomycetes taxa from underexploited or from unexplored habitats consider as very important sources of new bioactive compounds [11, 12]. Hence, this article would concentrate on the recent scenario about diverse actinomycetes and their secondary metabolites.

## Distribution of actinomycetes

**Fig. 1:**
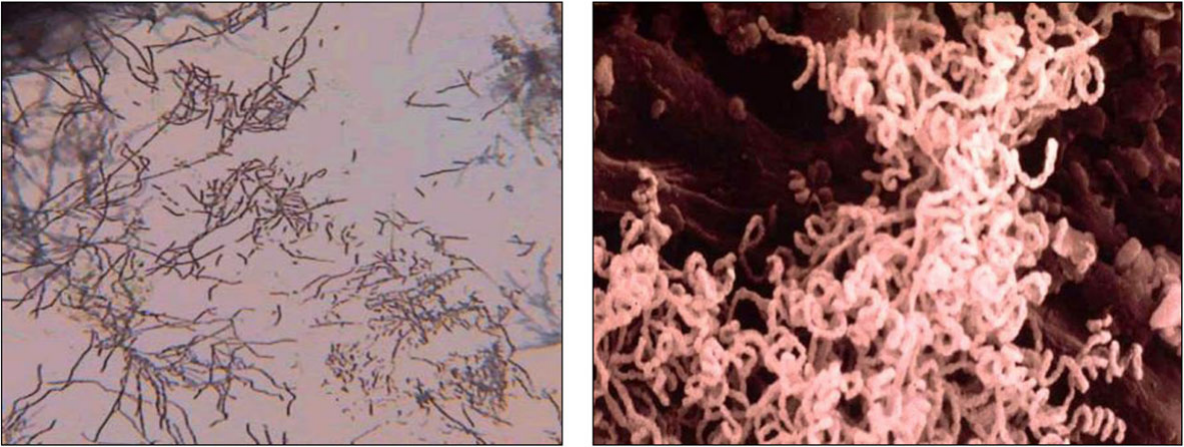
Aerial mycelium of *Streptomyces* sp.

**Fig. 2:**
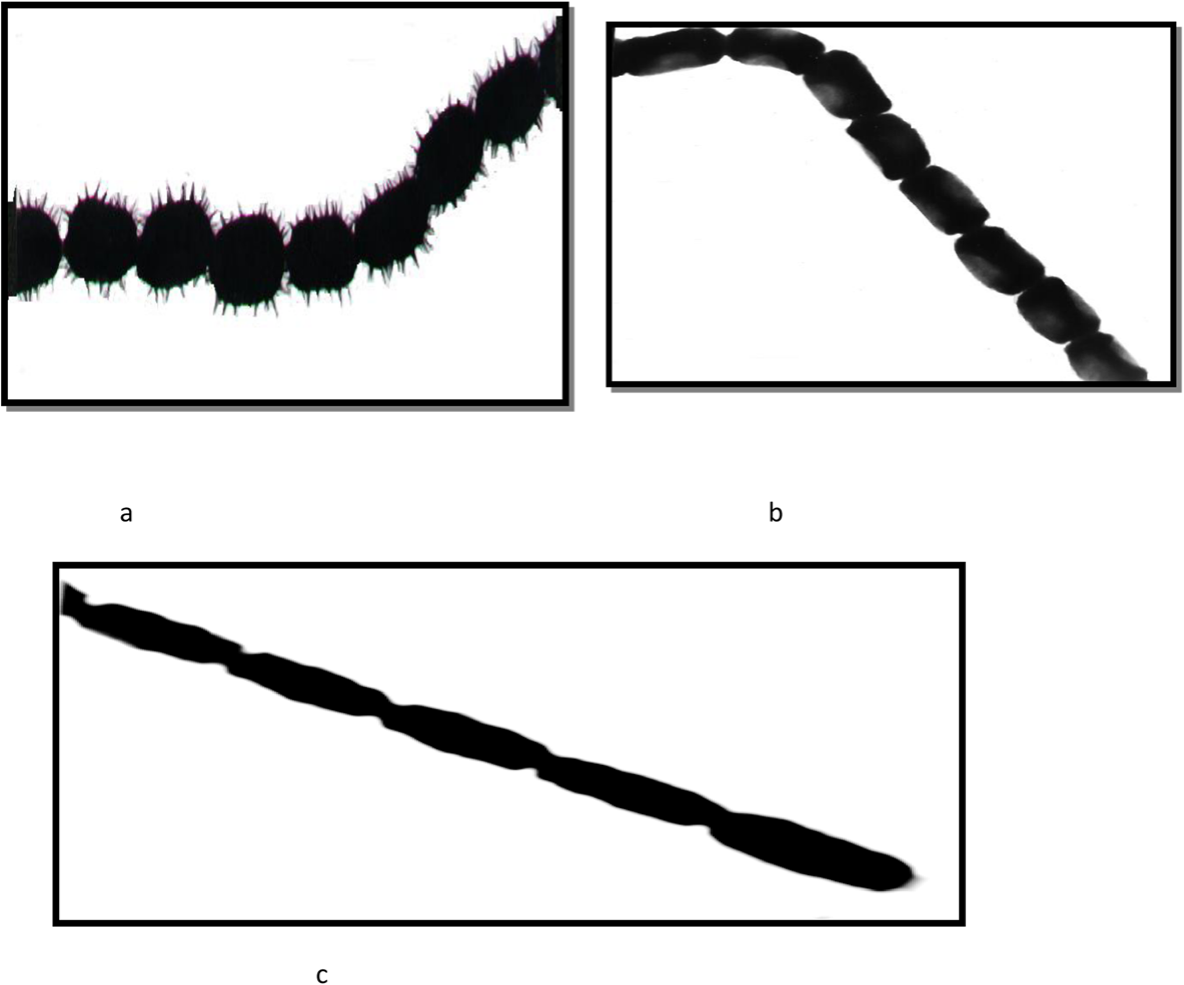
Different types of spore surface of *Streptomyces* sp. **a** (spiny), **b** (smooth), and **c** (warty)

Actinomycetes are capable of surviving in different habitats and are widely distributed in natural ecosystems. Actinomycetes are known as Gram +ve bacteria and characterized by the formation of aerial and substrate mycelium on solid media, also the presence of spores with different spore surfaces (Figs. 1 and 2), and high Guanine + Cytosine content of DNA [13]. Based on of morphological and chemical criteria, actinomycetes have been grouped into different genera (Table 1); *Streptomyces* is the most commonly isolated genera of the order Actinomycetales due to its great importance in medical science, ecology, and the biotechnology industry [11].

**Table 1:** Different groups of actinomycetes

Section	Characteristics
Nocardioformactinomycetes	Aerobic, may be acid-alcohol fast; occur as rods, cocci, and branched filaments or form substrate and aerial mycelium that fragment; wall chemotype IV; contain mycolic acids.
Actinomycetes with multilocular sporangia	Aerobic to facultatively anaerobic; mycelium divides in all planes, no aerial hyphae, wall chemotype III.
Actinoplanetes	Aerobic sporoactinomycetes, nonmotile, spores may be enclosed within vesicles; no aerial mycelium; wall chemotype II; whole-organism hydrolysates contain arabinose and xylose.
Streptomycetesand related genera	Aerobic sporoactinomycetes; form an extensively branched substrate and aerial mycelium.
Thermomonospora and relatedgenera	Aerobic spordactinomycetes; form an extensively branched substrate and aerial mycelium, both of which may carry single of chains of spores; spores either motile or non-motile; wall chemotype III
Thermoactinomycetes	The stable filaments produce aerial growth. Single spores (endospores) are formed on both aerial and vegetative filaments. All species are thermophilic. The cell wall contains meso-DAP but no characteristic amino acids or sugars.
Other genera	They all produce aerial growth-bearing chains of spores

*DAP* diaminopimelic acid

### Soil

Actinomycetes population has been known as soil inhabitant. It was stated that only 10% of the actinomycetes has been isolated from nature [14]. So the researchers need to screen more actinomycetes that have not been discovered and capable of producing new antibiotics active against bacteria that are resistant to current antibiotics [15].

### Rhizosphere soil

There are several groups of actinomycetes that are stable in bulk soil and in rhizosphere plants. Actinomycetes are very important for many plants, where rhizospheric streptomycetes can protect plant roots by inhibiting the growth of fungal pathogen a character based on their ability to produce antifungal antibiotics in vitro [16]. Due to their metabolic diversity, actinomycetes are considered a good source of lytic enzymes, antibiotics, and other bioactive metabolites.

### Actinmycorrhizal plants

These plants are characterized by formation in their roots nitrogen fixation nodules in combination with actinomycetes like *Frankia* sp. It was mentioned that a novel antibiotic called calcimycin was produced by *Frankia* sp. [17].

### Hypersaline soil

Hypersaline habitats are known as typical extreme environments which include saline lakes, saline soils and salterns. Hypersaline soils contain 9 to 23% salts. Examples of actinomycetes genera that isolated from hypersaline soils and showed antifungal activity against *Fusarium solani*, *Aspergillus niger*, and *Cryptococcus* sp. were *Streptomyces alboflavus*, *Nocardia* sp., *Micromonospora* sp., and *Streptomyces griseoflavus* [3, 11]. While some actinomycetes showed antibacterial activity against *Staphylococcus aureus* and *Escherichia coli* were *Streptomyces exfoliates*, *Streptomyces diasticus*, *Streptomyces albus*, and *Streptomyces albidoflavus* [18].

### Limestone

Limestone is different crystal form of CaCO3 and also known as a sedimentary rock which contains aragonite beside minerals calcite [19]. These hard conditions are very promising for isolation and screening for new isolates that produce novel bioactive compounds where *Streptomyces* sp. MBRL 10 showed antifungal activity against *Rhizoctonia solani* [20].

### Freshwater

It was known in recent years that the value of freshwater as a source of actinomycetes where genus *Streptomyces* was dominant in the river water while genus *Micromonospora* was dominated in the sediments of the river. These actinomycetes have antifungal activity from this habitat and it might be new sources for antimycotic agents against yeast and molds that affect patients with the terminal disease [21].

### Marine

The distribution of actinomycetes in the aquatic environment has been to a great extent undiscovered and the existence of primitive marine actinomycetes in the oceans stays a mystery. This is due to the absence of attempt in the discovering of marine actinomycetes taxa, while actinomycetes of terrestrial habitats have been, until lately, a useful source of new bioactive compound [22]. Actinomycetes founded in unusual marine conditions, for example in marine deep-sea gas hydrate reservoirs and organic aggregates, where they are the main components of the microbial communities. In the Wadden Sea, the actinomycetes isolated from the marine organic aggregates in it exhibited high antagonistic activity within this community. These marine actinobacteria yield valuable compounds are used in the pharmaceutical industry [23]. Bonafide actinomycetes are widely distributed in different marine ecosystems not only exist in the oceans [22]. It was stated that marine actinobacteria give a revelation of new classes of therapeutics that give the medications expected to support us for the following several years in our fight against drug-resistant infectious diseases [24]. Marine actinomycetes produced excessive antibiotics with diverse chemical structures. Actinomycetes account for more than 45% of all bioactive metabolites discovered in nature [25]. Marine actinomycetes are very important microorganisms because of their significant role in both biological and biotechnological applications. Till now, there are 83 species of actinomycetes that belongs to 28 genera that have been isolated from marine habitats and most of them are new to science. When more deep-sea studies are done, the diversity of the marine actinomycetes will be expanded [26]. About sixty-four of new secondary metabolites produced by marine streptomycetes demonstrate high antimicrobial and anticancer activities. Aside from this, diverse enzymes and enzyme inhibitors were revealed from the marine streptomycetes. Furthermore, a portion of these novel compounds after clinical investigation have not demonstrated any toxicity and side effects [27, 28].

### Sponges

As a noteworthy source of novel natural bioactive compounds, marine sponges harbor a lot of microbes in their tissues that can amount to forty percent of their biomass and it is widely believed that huge numbers of sponge’s products are produced by symbiotic microorganisms [29]. Marine sponges are one of the basic parts of deep-sea communities. They have developed specific relationships with various microorganisms and these relationships have ecological and biological importance. New actinomycete taxa have been detected in the Great Barrier Reef sponges, and also in the Mediterranean sponges where they produced different bioactive compounds [30].

### Volcanic cave-hot spot

Volcanic caves have been minimally concentrated for their potential as sources of new bioactive compounds and microbial species with new scaffolds. Volcanic cave microbiology from Canada recommends that this specific habitat has extraordinary potential for the isolation of new bioactive secondary substances. *Beutenbergia cavernae*, a new genus actinomycete and *Agromyces subbeticus* isolated from southern Spain cave [31], where these new isolates have antimicrobial activities against a variety of resistant pathogens from [32].

### Desert

The desert biome is viewed as a special, under-investigated source of novel actinobacterial diversity, with countless microorganisms found in soil samples got from hyper-arid areas of the Atacama Desert [33]. Because of large amounts of oxidation, the soil inside the extreme hyper-arid area is drained in natural material and comprises of low levels of culturable bacteria; so, the Yungay region of the Atacama Desert gives a promising setting to explore the survival of microorganisms in states of extraordinary aridity [34]. A significant number of these metabolites have antimicrobial activities and can be developed as therapeutic agents. It is additionally trusted that the desert soil may harbor an extensive populace of halophilic and alkaliphilic actinomycetes [35].

### Air

The ability of actinomycetes spores to be in the air was detected where air contains different types of their spores. It was stated that spores of airborne actinomycetes like *Nocardia* sp. are responsible for different antimicrobial production [36, 37].

### Insects gut

The digestive tracts of insects contain communities of symbiotic and transient microorganisms where these organisms provide novel bioactive microbial products [38]. Generally, insect gut microbiota provides a large addition to the nutrition host’s insect, as cleared examples like termites, cockroaches, and aphids. Honeybees, *Apis mellifera*, are a fascinating model for investigations of gut microorganisms due to the complex digestive tract. *Streptomyces* sp. sometimes could become the main organism in the bee guts. Species of *Nocardiopsis* were also identified in the gut of bee and characterized by the expression of an antibiotic biosynthetic gene [39]. The bioactive compounds produced by actinomycetes were specific against bee indigenous *Bacillus* strains furthermore two drug-resistant Gram +ve human pathogens. *Nocardiopsis alba* is a rare actinomycete isolated from the honeybee gut. Phenazine produced by *Nocardiopsis alba* could add to its capacity to temporarily survive under anaerobic conditions that occur in honeybee guts [40].

### Earthworm castings

**Fig. 3:**
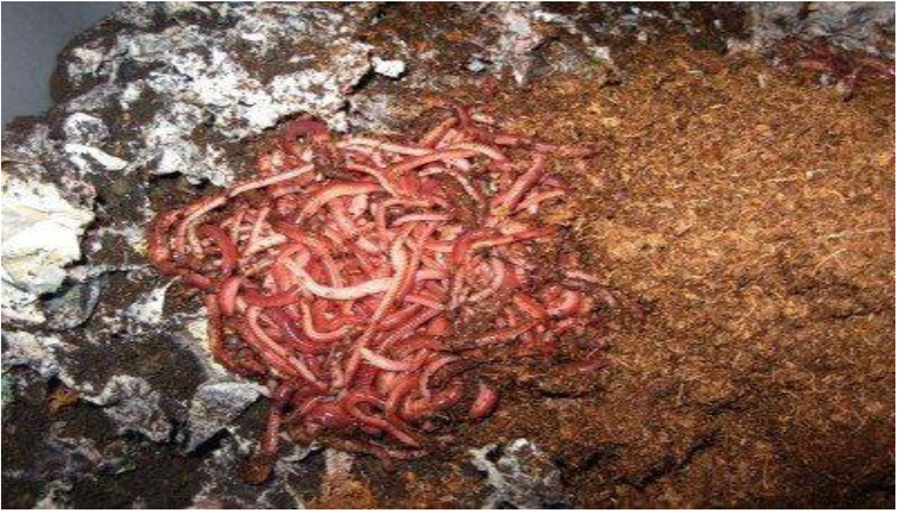
Earthworm casting

The earthworm casting (Fig. 3) has rarely been investigated for actinomycetes having antimicrobial activity and industrial enzymes. The casting activity led to nutrition and enrichment in soil. The earthworm redistributes organic matter within the soil, where it leads to an increase of the permeability and microbial activity by its burrowing and feeding activity. The dominant genera were *Streptomyces* followed by *Streptosporangium*. *Streptomyces* from casting was antagonistic to wood degrading fungi. Actinomycetes from casting have wide application in human and veterinary medicines respectively [41].

### Goat feces

Most actinobacteria are not obligate pathogens but true inhabitants of the environment. A symbiotic interaction with actinomycetes is essential for the survival and reproduction of many insects. Streptomycetes species appear to protect European beewolf offspring against infection by pathogens. In goat feces, the dominant species are *Oerskovia* and *Nocardiopsis*, where they produce larger numbers of antifungals than the antibacterial agent. Antibiotics like monensin and flavomycin produced by *Streptomyces* species have been used for growth—promoting in ruminants [42].

### Endophytic actinomycetes

Endophytes are microorganisms that live for the entire or part of their life history inside plant tissues [43]. Because of these long held affiliations, endophytic microorganisms and plants have grown great information exchange. Endophytic actinomycete produced bioactive compounds were used as biocontrol agents like compounds produced by *Nocardia globerula* to control *Helminthosporium solani* pathogen which causes silver scurf illness in potato.

*Streptomycetes* sp. compounds showed antifungal activity. Ansacarbamitocins were separated from actinomycetes strain *Amycolatopsis* CP2808 which has a place with family pseudonocardiaceae. Ansamitocin is a group of ansamycin antibiotics that demonstrates strong antitumor action [44]. Ansamitocin was produced by endophytic actinomycetes *Nocardia* sp.

## Secondary metabolites from actinomycetes

Secondary metabolites created by Actinomycetes display an incredible number of diverse biological effects, besides the antimicrobial activities. The order Actinomycetales are responsible for the production of bioactive compounds with a highly record of more than 10,000 antimicrobial agents in pharmaceutical use (Table 2) [45].

**Table 2:** Numbers of actinomycetales species, including the all rare actinos, known to produce bioactive metabolites, are summarized [45]

Actinomycetales species	No.	Actinomycetales species	No.
**Streptomycetaceae**		**Thermomonosporaceae**	
Streptomyces	8000	Actinomadura	345
Streptoverlicillium	258	Saccharothrix	68
Kitasatosporia	37	Microbispora	54
Chainia	30	Actinosynnema	51
Microellobosporia	11	Nocardiopsis	41
Nocardioides	9	Microtetraspora/Nonomuria	26/21
**Micromonosporaceae (Actinoplanetes)**		Thermomonospora	19
Micromonospora	740	Micropolyspora/Faenia	13/3
Actinoplanes	248	Thermoactinomyces	14
Dactylosporangium	58	Thermopolyspora	1
Ampullariella	9	Thermoactinopolyspora	1
Glycomyces	2	**Mycobacteriaceae (Actinobacteria)**	
Catenuloplanes	3	Nocardia	357
Catellatospora	1	Mycobacterium	57
**Pseudonocardiaceae**		Arthrobacter	25
Saccharopolyspora	131	Brevibacterium	17
Amycalotopsis/Nocardia	120/357	Proactinomyces	14
Kibdellosporangium	34	Rhodococcus	13
Pseudonocardia	27	**Other (unclassified) species**	
Amycolata	12	Actinosporangium	30
Saccharomonospora	2	Microellobosporia	11
Actinopolyspora	1	Frankia	7
**Streptosporangiaceae (Maduromycetes)**		Westerdykella	6
Streptosporangium	79	Kitasatoa	5
Streptoalloteichus	48	Synnenomyces	4
Spirillospora	11	Sebekia	3
Planobispora	10	Elaktomyces	3
Kutzneria	4	Excelsospora	3
Planomonospora	2	Waksmania	3
		Alkalomyces 1	1
		Catellatospora 1	1
		Erythrosporangium	1
		Streptoplanospora	1
		Microechinospora	1
		Salinospora	1

### Antibiotics

**Fig. 4:**
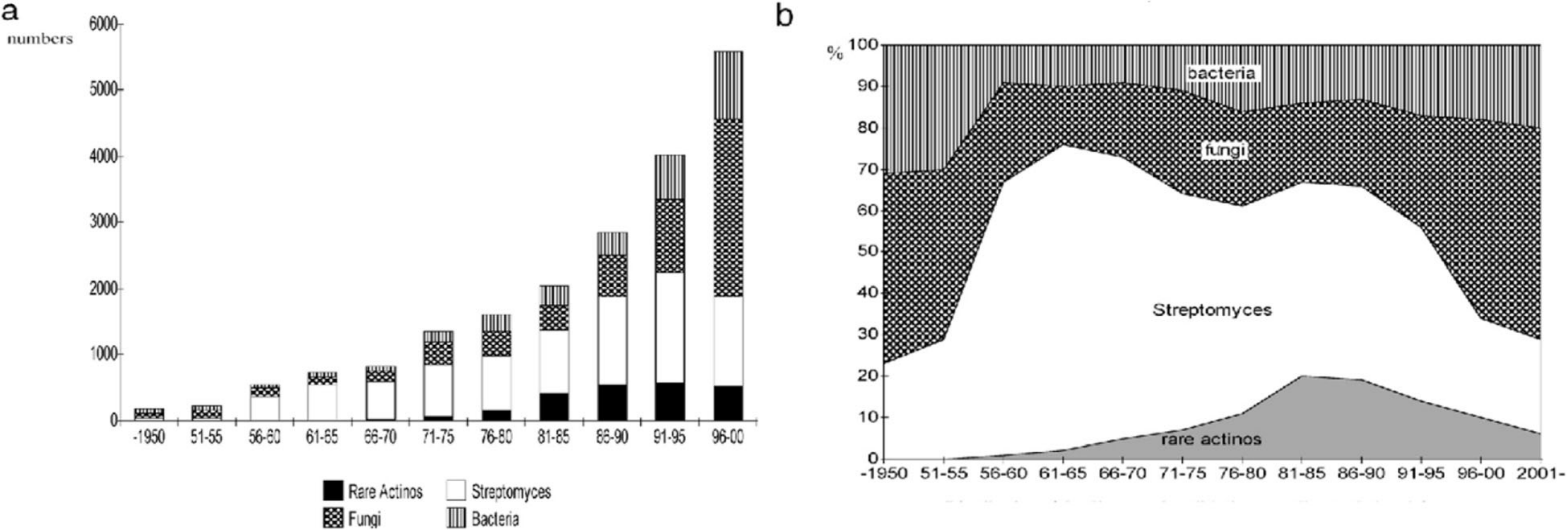
Distribution of the discovered antibiotics according to their origin **a** (number), and **b** (percentage) [48]

Antibiotics are an exceptional sort of chemotherapeutic agent produced by living organisms; bacteria, fungi, and actinomycetes, which in little amounts can hinder the growth of microorganisms or even kill them. Antibiotics are defined as low molecular weight organic compounds produced by microorganisms [46]. About seventy-five percent of antibiotics mainly antibacterials are produced by actinomycetes. Huge numbers of antibacterials show an expansive range of activities and various systems of activity. They demonstrated high intensity against a huge number of Gram +ve and Gram −ve bacteria [47]. Historically, the origin of the biggest number of the new antibiotic drugs was streptomycetes when comparing with bacteria and fungi (Fig.4) [45, 48]. This order alone created about 45% of the known bioactive metabolites; more than ten thousand compounds were isolated from different actinomycetales species, about thirty-four percent from *Streptomyces* and eleven percent from the other actinomycetes [49].

Disclosure of new antibiotics secreted by streptomycetes continue for instance; mediomycins A, B, and clethramycin, were isolated from *Streptomyces mediocidicus* ATCC23936 and *Streptomyces malaysiensis* DSM4137 respectively show a wide spectrum of antifungal activity [50].

Polyketides are very important natural products because of their pharmaceutical applications. Instances of such polyketides are erythromycin (antibacterial), nystatin (antifungal), furthermore avermectin (antiparasitic). All the previous antibiotics have been produced by *Streptomyces* sp. which are considered as the principle producers of antibiotics (Table 3) [47].

**Table 3:** List of some antibiotics produced by *Streptomyces* sp*.* [47]

*Streptomyces* sp.	Antibiotic	*Streptomyces* sp.	Antibiotic
*S. orchidaccus*	Cycloserin n	*S. erythraeus*	Erythromycin
*S. oriantalis*	Vancomycin	*S. vensuella*	Chloramphenicol
*S. fradiae*	Neomycin, actinomycin, fosfomycin, dekamycin	*S. aureofaciens*	Chlortetracycline, dimethylchlor
*S. nodosus*	Amphotricin B	*S. ambofaciens*	Spiramycin
*S. noursei*	Nistatin	*S. avermitilis*	Avermicin
*S. mediterranei*	Rifampin	*S. alboniger*	Puromycin
*S. griseus*	Streptomycin	*S. niveus*	Novobicin
*S. knanamyceticus*	Kanamycin	*S. platensis*	Platenmycin
*S. tenebrarius*	Tobramycin	*S. roseosporus*	Daptomycin
*S. spectabilis*	Spectinomycin	*S. ribosidificus*	Ribostamycin
*S. viridifaciens*	Tetracycline	*S. garyphalus*	Cycloserine
*S. lincolensis*	Lincomycin, clindamycin	*S. vinaceus*	Viomycin
*S. rimosus*	Oxytetracyclin	*S. clavuligerus*	Cephalosporin

### Biopesticide agents

Microorganisms including which are antagonistic to insects are used to naturally control them. Actinomycetes produce insecticidal active compounds used in biological control of house fly *Musca domestica* [51]. The death of larval and pupal stages were high coming to up to ninety percent after using actinomycetes insecticide [52]. Actinomycetes were adequately used for controlling mosquito *Culex quinquefasciatus* [45, 47].

### Plant growth hormone

Actinomycetes have been used for the improvement plant growth by the production of plant growth hormones for example auxins and gibberellin-like compounds [52, 53]. Actinomycetes produced Indole-3-acetic acid (IAA) which is the main form of auxin; this compound is responsible for cell division, elongation, and differentiation.

### Antitumor compounds

Streptomycetes are differentiated groups that can create distinctive cytotoxic compounds which have anticancer activity [54–56]. The produced compound in Table 4 showed significant activity against different malignant cell lines [41]. Mitomycin is a natural product that contains different functional groups, like aminobenzoquinone and aziridine ring system. Mitomycin C (MC) was produced by *Streptomyces lavendulae* and has been generally utilized clinically for antitumor treatment [57, 58].

**Table 4:** List of the bioactive compounds derived from actinobacteria showed antibacterial and antitumor activities [41]

Bioactive compound	Species	Activity
Abyssomicin	*Verrucosispora* sp*.*	Antibacterial and antitumor
Actinofuranones A-B	*Streptomyces* sp.	Antibacterial and antitumor
Analogs-metacycloprodigiosin	*Saccharopolyspora* sp.	Anticancer
Benzanthraaquinone	*Chainia purpurogena*	Antibacterial and antitumor
Butenolides	*Streptomyces* sp.	Antitumor
Mechercharmycin	*Thermoactinomyces* sp.	Antitumor
Diphosphatidylglycerol	*Micromonospora* sp.	Antitumor

### Antiviral agents

*Streptomyces lavendulae* produced complestatins which are known as peptides. They did not show inhibitory activity against HIV enzymes, where these peptides act by the interaction with the cell surface molecules of the target cells and inhibiting the adsorption of human immunodeficiency virus type 1 (HIV-1) to the cells [59] (Table 4). A list of the bioactive compounds derived from actinobacteria showed antibacterial and antitumor activities [41].

*Streptomyces chromofuscus* produced protease inhibitor (PISC-2002) from culture supernatants of it. PISC-2002 plays an important role as an antiviral agent against influenza virus A/Rostock/34 (H7N7) [60]. *Streptomyces* sp. produced pimprinine, which is an extracellular alkaloid. Pimprinine shows significant physicochemical properties, antimicrobial activities, anticonvulsant activity and also shows antiviral activity against Enterovirus 71 (EV71) [61].

### Pharmacological compounds

Vitamin B12 is a very important vitamin of the B complex (B1, B2, B3, B6, and folic acid) group. It was first recorded in 1948 from *Streptomyces griseus*. There is no source of this vitamin except microbial synthesis [62].

### Pigments

**Fig. 5:**
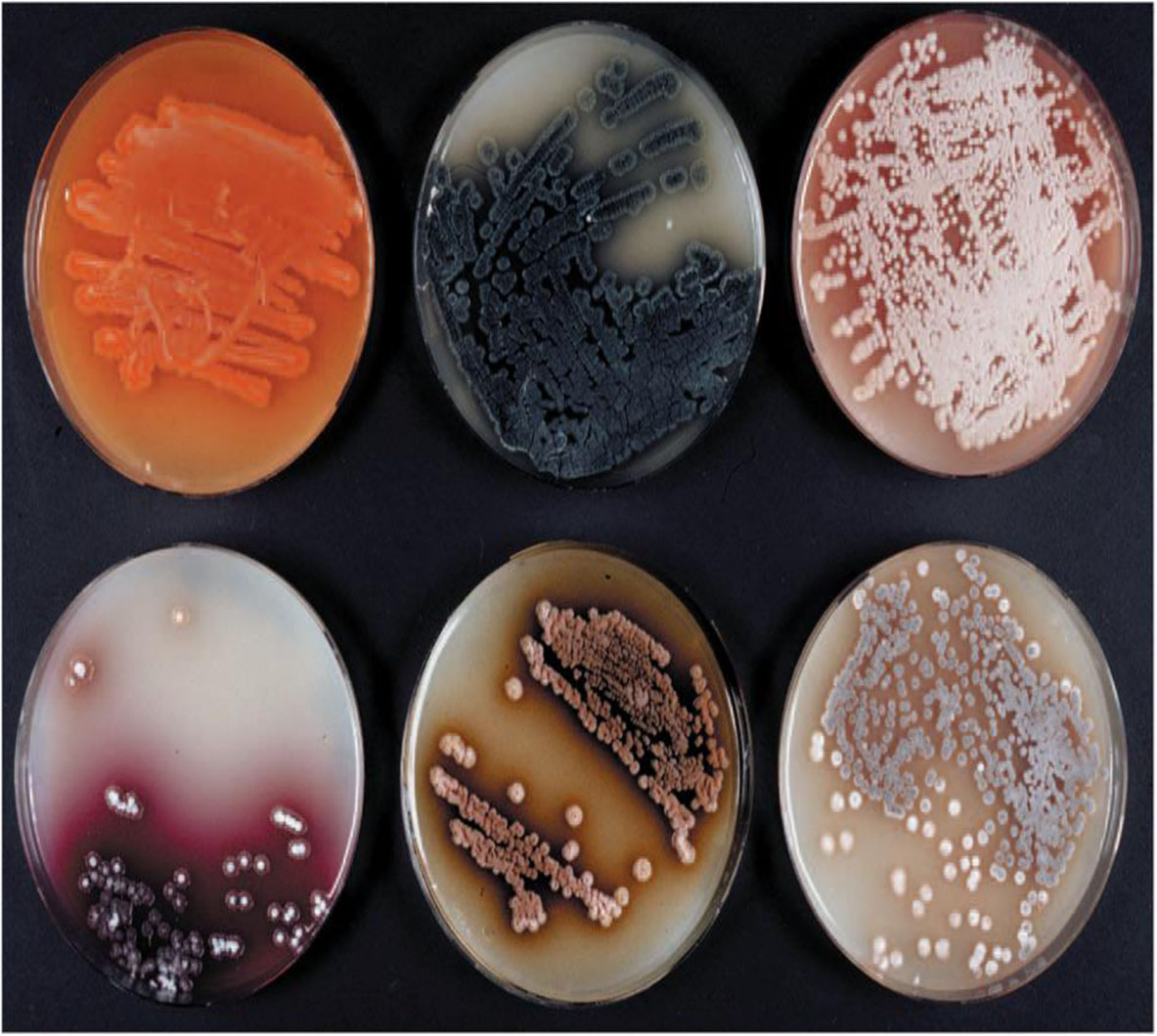
Production of pigmented secondary metabolites by *Streptomyces* colonies [63]

**Fig. 6:**
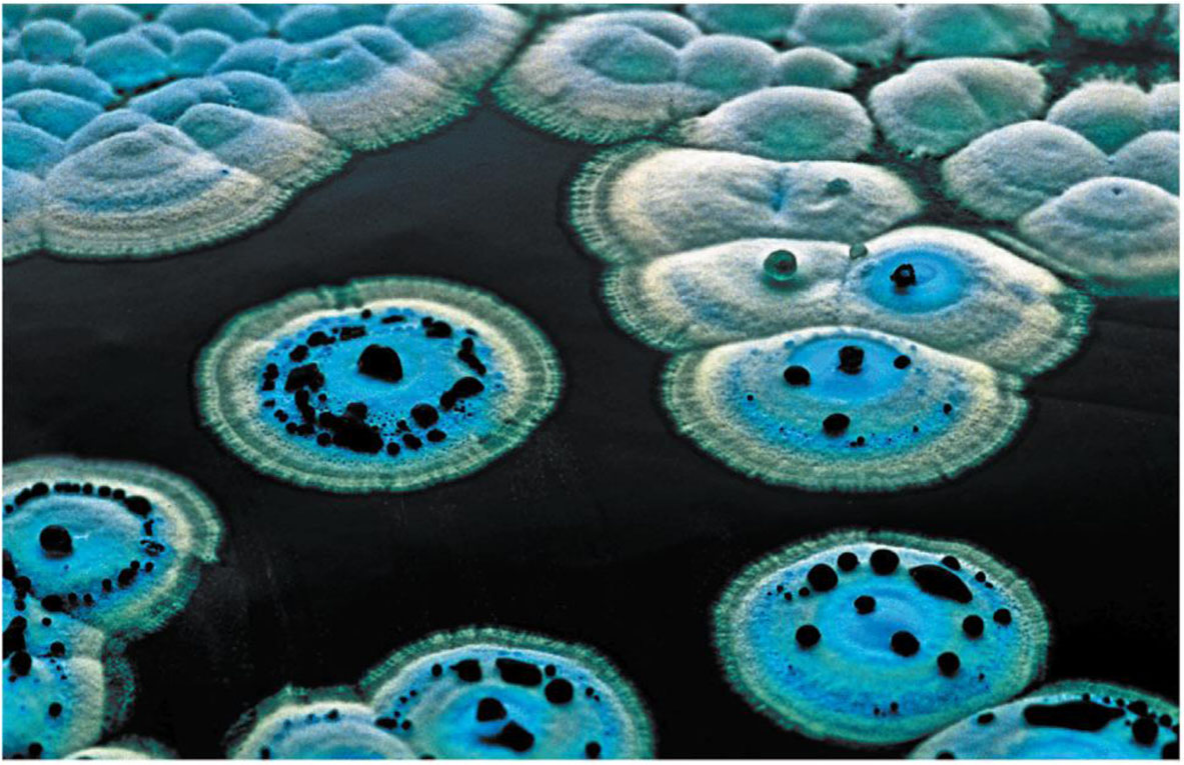
A panoramic view of *Streptomyces coelicolor* including the blue antibiotic actinorhodin is excreted into the medium and into aqueous droplets on the hydrophobic surface of the colony [63]

Actinomycetes are characterized by the different production of pigments on natural or synthetic media. These pigments are usually blue, violet, red, rose, yellow, green, brown, and black. The pigments might be diffuse into the medium or they might be retained in the mycelium (Fig. 5) [63]. Actinomycetes had known to be produced different sorts of antibiotics, and additionally, these antibiotics include numerous pigments [64]. Melanins are regularly used in pharmacology and cosmetics. *Streptomyces virginiae* produced the highest level of pigment in peptone-yeast extract-iron followed by tyrosine liquid medium. The pigment-producing actinomycete *Streptomyces hygroscopicus* showed antibacterial activity against many drug-resistant pathogens like methicillin-resistant *Staphylococcus aureus* (MRSA), vancomycin-resistant *Staphylococcus aureus* (VRSA), and extended-spectrum β-lactamases (ESBL) strains. *Streptomyces* species produced yellowish antibiotic pigment 4-hydroxynitrobenzene. Where the yellow pigment was extracted in chloroform and showed activity against *Bacillus subtilis* and *Shigella shiga*. Microbial pigments have antibiotic or anticancer activities and are also safe for human utilization. Few of them are also demonstrated as nourishment grade pigments. They are easy to produce and are economic too [65, 66]. Streptomycetes is a major industrially important class, which have the ability to produce numerous antibiotics and pigments. The ability of these organisms to produce pigments depends on the various conditions of nutrition and cultivation where it can be greatly increased or completely lost. So, it is very important to improve the right combination of different cultural conditions to encourage growth and pigment production. A biological pigment actinorhodin is produced by *Streptomyces coelicolor*, *Streptomyces violaceusruber*, and *Streptomyces lividans*. It is blue in color and based on the pH (Fig. 6) [67]. This pigment has different application such as an antibiotic against Gram +ve bacteria, an indicator in laboratory agents because of its ability to show special colors in acid and alkali medium and finally, actinorhodin can be used in the food industry during making beverages, desserts, etc. and might be even in cosmetic industry. The complete scope of pigment’s application has not been yet explored [68].

### Commercial enzymes

The value of commercial enzymes has been expanded considerably with their different uses in pharmaceutical, food, and detergents industry. Actinomycetes have a different active enzyme which is able to catalyze different biochemical reactions with new enzymes. The applications of few commercial enzymes are listed in Table 5 [41].

**Table 5:** Commercial enzymes produced by actinomycetes with their application [41]

Enzyme	Actinomycetes strains	Use	Industrial of application
Protease	*S. galbus*	Detergents	Detergents
		Cheese making	Food
		Clarication—low calorie beer	Brewing
		Dehiding	Leather
		Treatment of blood clot	Medicine
Cellulase	*S. actuosus*	Removal of stains, denim nishing, soening of detergent	Deinking, modication of bers, paper and pulp Denim nishing, soening of cotton
Lipase	*S. griseochromogenes*	Removal of stains	Detergent
		Stability of dough and conditioning	Baking
		Cheese avoring	Dairy
		Deinking, cleaning	Textile
Xylanase	*S. rameus*	Conditioning of dough	Baking
		Digestibility	Animal feed
		Bleach boosting	Paper and pulp
Pectinase	*S. fradzae, S nztrosporeur*	Clarication, mashing	Beverage
Amylase	*S. aureofasciculus, S. galilaeus*	Soness of bread soness	Detergent
		Removal of stains volume	Baking
		Deinking, drainage improvement	Paper and pulp
		Production of glucose and fructose syrups	Starch industry
		Removal of starch from woven fabrics	Textile
Glucos oxidase	*Streptomyces* sp.	Strengthening of dough	Baking
Lipoxygenase	*Streptomyces* sp.	Bread whitening	Baking
Phytase	*S. ambofaciens, S. lienomycini.*	Phytate digestibility	Animal feed
Peroxidase	*Thermomonospora fusca, S. viridosporus*	Removal of excess dye	Textile
β-galactosidase	*Streptomyces* sp.	Enzymatic hydrolysis of lactose either from milk/whey or pure lactose	Dairy
l-asparaginase	*S. aureofasciculus, S. canus, S. chattanoogenesis, S. hawaiiensis, S. olivoviridid, S. orientalis, S. plicatus*	Reduce the formation of acrylamide, a carcinogen found in starchy food products	Food industry
l-glutaminase	*S. rimosus, S. galbus*	Flavor enhancing agent in food	Food industry
Keratinase	*Doretomycetes microsporus*	Animal feed	Poultry industry
Petinase	*Thermomonospora flisca S. viridochromogenes*	Retting and degumming of fiber crops	Textile industry

**Fig. 7:**
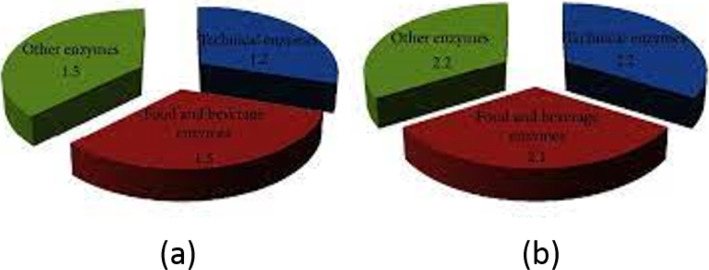
Global enzyme industry market in the years **a** (2011), and **b** (2016) [72]

**Fig. 8:**
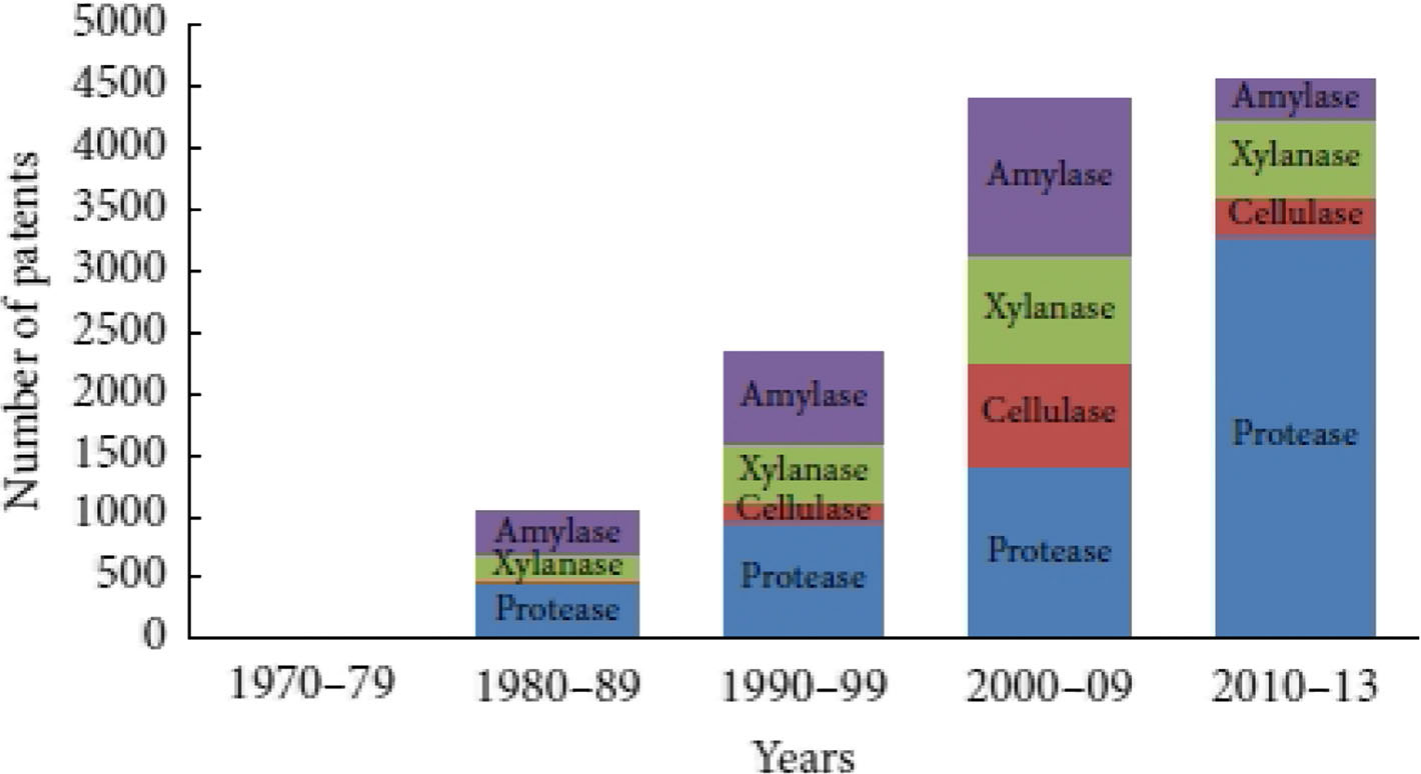
Growth in number of patents issued for important industrial enzymes over past few decades [72]

*Streptomyces* species produced important enzymes like amylase, protease, and cellulose which have commercial applications in different industries [69]. l-glutaminase, l-asparaginase, and α-galactosidase have an effective role in biocycling of carbon and nitrogen in natural water and sediments. l-glutaminase and l-asparaginase showed antitumor activities and were produced by marine streptomycetes [70, 71]. The global industrial enzyme market has been increased continuously because of various mergers and acquisitions (Fig. 7). Food and beverage enzymes have great attention in these decades. There are additional increments in patent number in the decades in straight scale which are given in Fig. 8 [72].

### Enzyme inhibitors

Enzyme inhibitors have gotten expanding consideration as valuable tools, not just for the study of enzyme structures and reaction mechanisms yet additionally for potential usage in the pharmacological industry [73]. Marine actinomycetes are promising sources for enzyme inhibitor production [74, 75], it was reported that different types of enzyme inhibitors like N-acetyl--d-glucosaminidase, pyroglutamyl peptidase, and α-amylase inhibitors are produced from marine actinomycetes. Amylase inhibitors are starch blocker since they contain substances that keep dietary starches from being consumed by the body. The inhibitors might be helpful for weight loss.

Alpha-amylase inhibitors are produced by *Streptomyces corchorushii* and *Streptomyces* sp. CC5 respectively [75]. *Streptomyces toxytricini* produced lipstatin, which is a very exceptional inhibitor of pancreatic lipase [76].

### Anti-inflammatory compounds

Saphenic acid and lipomycin were found to have anti-inflammatory activity that is produced by marine actinomycetes [77]. Also, it was stated that *Micromonospora* sp. produced bioactive compounds with anti-inflammatory activity along with antimicrobial activity [78]. *Streptomyces arenicola* also produces the anti-inflammatory metabolites cyclomarin A and C, where it was reported that cyclomarin A has anti-tuberculosis and anti-malaria activity [79]. It has been noticed that swelling was decreased when cyclomarin A was managed topically or intraperitoneally [80].

### Single-cell protein feed

Marine actinomycetes can be utilized as fishmeal. Actinomycetes can produce some secondary metabolites which may improve the development of young fish and shrimp. Some of these metabolites for example boromycin, aplasmomycin [81], and regular amino acids like alanosine, amino dichlobutyric acid, azaleucine, and 4-oxalysine [82]. Young prawns and shrimps fed on actinomycetes incorporated feed indicated a higher development rate, more food conversion efficiency, and higher protein content [82]. So, among the unusual protein sources, single-cell protein of microbial origin seems to be a promising substitute for fishmeal, which can supplant up to 25 to 50% fishmeal in aquaculture operations.

### Biosurfactant

A biosurfactant is a surface-active molecule produced mainly by microorganisms. The term refers to compounds having some influence on interfaces. The evaluation of biosurfactants is carried out through surface tension measurements. In the literature, the terms surfactant and emulsifier are frequently used interchangeably [83, 84].

Biosurfactants have numerous advantages due to their specificity, biodegradability, and less toxicity. Also, the effectiveness of these biosurfactants to work under extreme conditions of temperature, pH, and salinity. Actinomycetes play an important vital role in the production of bioemulsifiers. Trehalose dimycolates produced by certain actinomycetes like *Nocardia* sp. [85]. Also, *Streptomyces griseoflavus* and *Nocardiopsis* A17 were produced numerous biosurfactants [86].

## Conclusion

The utilization of Actinomycetes as a hotspot for new bioactive optional metabolites in its earliest stages. The antagonistic Actinomycetes evidence that the ecosystem is an important source of biologically active secondary metabolites. Finding powerful secondary metabolite producers like Actinomycetes is an interesting and challenging platform for scientists. Despite of growing under extraordinary conditions, members of Actinomycetes produce industrially valuable compounds, for example, enzymes, antibiotics, and pigments. The various ecological habitats demonstrate the existence of Actinomycetes species in specific microbial niches. However, the ecological habitat is underexplored and yet to be investigated for unknown, rare Actinomycetes diversity.

## Data Availability

Not applicable
